# Nonlinear phenomena in mammalian vocal communication: an introduction and scoping review

**DOI:** 10.1098/rstb.2024.0017

**Published:** 2025-04-03

**Authors:** Jen Muir, Christian T. Herbst, Joseph E. Hawes, Thomas O’Mahoney, Jacob C. Dunn

**Affiliations:** ^1^ Behavioural Ecology Research Group, School of Life Science, Anglia Ruskin University, Cambridge CB1 1PT, UK; ^2^ Department of Behavioral and Cognitive Biology, University of Vienna, Vienna 1030, Austria; ^3^ Department of Communication Sciences and Disorders, College of Liberal Arts and Sciences, University of Iowa, Iowa City, Iowa 52240, USA; ^4^ Institute of Science and Environment, University of Cumbria, Ambleside, Cumbria LA22 9BB, UK; ^5^ ENES Bioacoustics Research Laboratory, University of Saint Étienne, St-Étienne 42023, France

**Keywords:** vocal production, bioacoustics, biphonation, chaos, frequency jump, subharmonics

## Abstract

Nonlinear phenomena (NLP) are common elements of mammalian vocalizations. Resulting from irregular sound production, they contribute to perceived harshness and are often present in calls conveying urgency or arousal. Initially dismissed as by-products of vocal production, NLP are increasingly recognized for their adaptive potential. However, NLP have never been the subject of a comprehensive review across vertebrate taxa. Here, we introduce NLP and examine developments in NLP studies in mammals. We found 220 papers published between 1962 and 2023, with publication rates increasing with time. The studies covered a wide range of taxonomic groups but were dominated by artiodactyls, carnivores, bats, rodents and primates. Tinbergen’s questions offer a framework for future investigations, highlighting that while much research has been conducted on adaptive function, our understanding is still lacking in terms of ontogeny, mechanisms and evolution. The existing literature is a testimony to the importance of NLP in animal vocalizations. With the use of novel tools for analysis and playback studies, NLP research can become more cohesive and impactful, fostering better understanding among researchers. We look forward to a new age of NLP research, which we anticipate will lead to a paradigm shift in our understanding of vocal communication in mammals.

This article is part of the theme issue ‘Nonlinear phenomena in vertebrate vocalizations: mechanisms and communicative functions’.

## 1. Introduction

Vocal communication represents a key element of the behaviour of many vertebrate species, strongly linked to reproduction and survival [1–4]. Given this, there is a long history of research into the biology, ecology and evolution of vocal communication across a wide range of vertebrate taxa [1]. Early discussions of animal vocalizations distinguished harmonic, tonal calls (e.g. bat chirps [5] and birdsong [1]), from perceptually harsh calls (e.g. howler monkey, *Alouatta* spp., roars [6]; red deer, *Cervus elaphus*, roars [7]). With advances in the study of vocal production [8,9] and the addition of dynamical systems theory [10,11], such perceived harshness was shown to be due to the presence of acoustic irregularities termed ‘nonlinear phenomena’ (NLP) within calls.

NLP were initially studied in human vocalizations—mainly in infant cries and in those with vocal disorders [12,13]. However, they have since become a growing subject of interest in bioacoustics and studies have expanded into mammals [14], birds [15], herptiles [16] and fish [17]. Despite this growing literature, NLP have never been the subject of a comprehensive review across vertebrate taxa, and the only review of NLP in mammals was published >20 years ago [18]. Thus, there is a need for an update of the status of this emerging field.

Here, we provide an introduction to NLP for the non-expert and a scoping review of the literature in this field since the first publications in the early 1960s. We chose mammals as the focus of this review owing to the large number of studies that have been published in this class (*n* = 220) compared to other vertebrate taxa (herptiles *n* = 23; birds *n* = 20; fish *n* = 2) and to simplify discussions of the mechanisms of vocal production, which vary across clades [19]. Notably, there is a considerable body of literature on NLP in the human voice, particularly in the context of pathological voice disorders [20]. However, we excluded humans here in order to (i) focus on the evolution and ecology of NLP in non-human animals, (ii) avoid the literature on NLP in voice pathology, and (iii) sidestep differences in terminology that have traditionally been used in human voice science, which would likely lead to unnecessary confusion.

## 2. An introduction to nonlinear phenomena in mammalian vocal communication

Voice production in vertebrates is governed by both the MyoElastic-AeroDynamic theory of phonation (MEAD) and Source Filter Theory (SFT) [21,22]. MEAD explains the generation of the self-sustaining acoustic voice source via fluid–structure interactions between a pulmonary airstream and vibrating laryngeal tissue. SFT explains how the resultant voice source is transmitted through, and acoustically filtered by, the vocal tract resonator and radiated into the environment. The quality of the sound generated by any mammal is thus governed by features of both the laryngeal voice source (larynx) and the vocal tract resonator (vocal tract).

Across mammalian species, the laryngeal sound generator, i.e. the vocal folds, can undergo tissue oscillations across an extraordinarily large range, from <10 Hz to >150 kHz [23,24], that is, more than four orders of magnitude. One fundamental property of the generated sound is its periodicity (regularity), or the lack thereof. A time series generated by an oscillating system is periodic if it repeats itself at a precisely fixed interval, satisfying the condition *x*[*t*] = *x*[*t* + *nT*], where *x* is the time series in question, *t* is the passing time, *T* is the period of oscillation (the interval at which the oscillation repeats itself, i.e. the duration of the glottal cycle) and *n* is an arbitrary integer. Such a condition of strict periodicity cannot be achieved by biological signals, because they typically contain (random) fluctuations [25]. For that reason, the voice can at best be described as nearly periodic (note that some texts alternatively but incorrectly use the term ‘quasi-periodic’ for this condition—see below). A voice signal can, however, considerably deviate from near periodicity, owing to NLP, and examples can easily be found in crying human babies, the pathological human voice, human singing and animal vocalizations.

Mechanistically, NLP arise within the laryngeal sound generator as deviations from periodicity or abrupt changes between different oscillatory states. These are caused by biomechanical properties of the vocal folds, potentially interacting with other laryngeal oscillator tissue (e.g. the ventricular folds) or with either the supraglottal and/or the subglottal vocal tracts [26,27].

**Figure 1.**
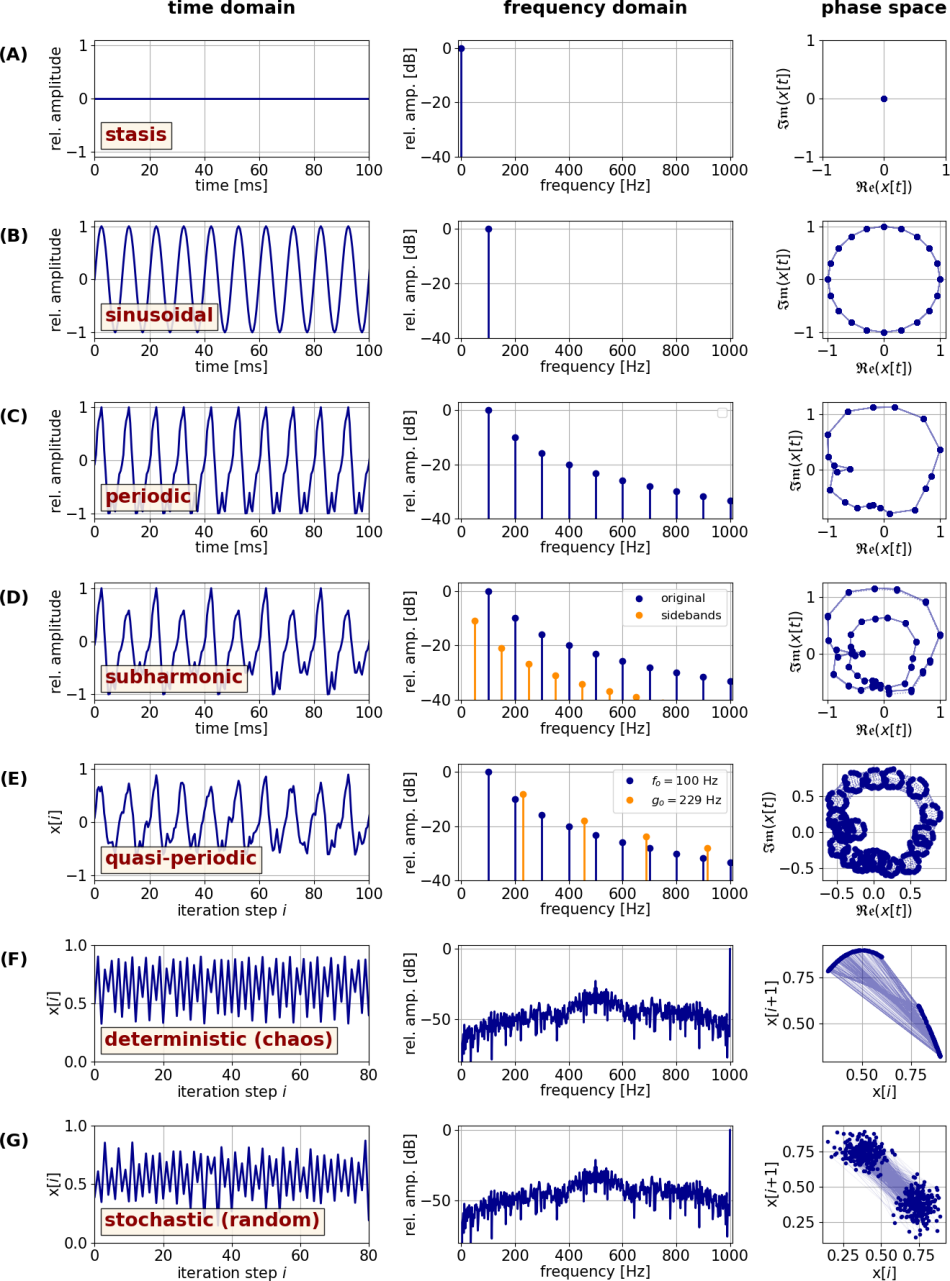
Prototypical oscillatory states of a dynamical system: (A) stasis, i.e. no vibration; (B) sinusoidal vibration; (C) cyclic oscillation, exhibiting a harmonic series; (D) subharmonic oscillation; (E) quasi-periodic signal with two harmonic series with incommensurable fundamental frequencies (arbitrarily chosen here); (F) irregular oscillation, established by deterministic (i.e. chaotic) system behaviour; (G) irregular oscillation, established by a stochastic (i.e. random) process. The three columns of the figure show an exemplary time series waveform (left) and the resulting frequency spectrum (middle) and phase space representation (right). The phase space representations for A through E were generated by applying a Hilbert transform to the respective time series [34]. The resulting analytic signal is made up of a real and an imaginary part of the signal, where the imaginary part contains all frequencies of the real component, but each frequency component is delayed by 90 degrees. In the phase space reconstruction, the real and the imaginary parts were plotted against each other. The phase space embeddings for F and G were generated by plotting the respective time series against a delayed version of itself, using a delay of one sample. The figure was algorithmically generated with Python code written by CT Herbst (available at https://www.christian-herbst.org/software/NLP/).

**Figure 2.**
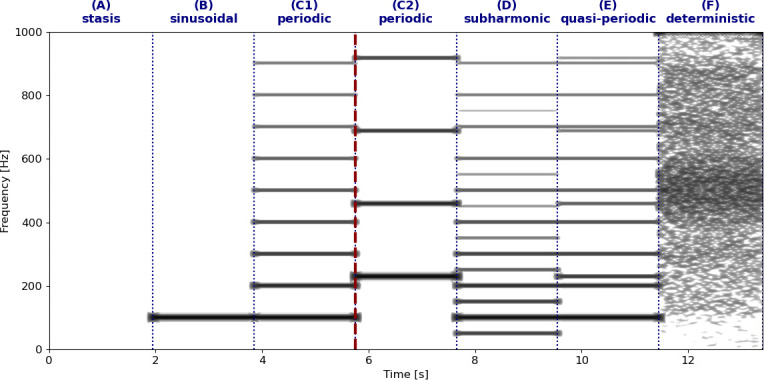
Narrow-band spectrogram visualization of the different oscillatory states shown in figure 1. Note that C1 and C2 both represent periodic signals with a different *f*_*o*_ and thus different harmonic series. The frequency jump bifurcation between C1 (*f*_*o*_ = 100 Hz) and C2 (*f*_*o*_ = 227 Hz) is indicated with a dark red dashed vertical line. The figure was algorithmically generated with Python code written by CT Herbst (available at https://www.christian-herbst.org/software/NLP/).

The notion of periodicity, and deviations thereof, was first described in nonlinear system dynamics [28,29], then introduced to acoustics [30] and later integrated into voice science [13,31–33]. NLP are deviations from periodicity in an acoustic signal, and as such it is useful to first introduce the typical oscillatory states that characterize vocal production by the sound source—the larynx. When assessing the behaviour of a dynamical system, the prototypical oscillatory states described below, and illustrated in figures 1 and 2, are of relevance (see also [27]):

(A) **Stasis**, i.e. a time-invariant system output, with the absence of any vibration. This is depicted in figure 1A, in which the time domain is represented by a waveform that has a constant value of 0.5 (left column). The resulting spectrum in the frequency domain (middle column) only contains a so-called direct current component at zero Hertz—no energy is found at any frequency greater than zero (i.e. in the alternating current region). Due to the lack of oscillation, the phase space [29] representation (right column) only emerges as one single point.
(B) A **sinusoidal**—and thus perfectly periodic—oscillation with a fundamental frequency (*f_o_*) of 100 Hz is depicted in figure 1B. In the frequency domain spectrum, exactly one component is visible, indicating the presence of oscillatory energy at 100 Hz. The phase space representation depicts a so-called limit cycle attractor (i.e. a closed trajectory in phase space to which all neighbouring trajectories converge over time [29, p. 324], which is exactly circular. Conceptually, the rotating phase space trajectory can be treated as a rotating phasor [35], allowing for the original time series to be reconstructed as a one-dimensional projection of the phase space data’s real part. Note that a precisely sinusoidal signal (i.e. sine wave) does not occur in human or vertebrate vocalization.
(C) A more realistic, **periodic** example—approximating the laryngeal voice source of modal phonation in humans with a spectral slope of −10 dB per octave [36]—is shown in figure 1C. The time-domain waveform (*f_o_* = 100 Hz) is cyclical and periodic, but not precisely sinusoidal because the signal contains higher frequency components at integer multiples of *f_o_*, establishing a so-called harmonic series. Owing to the harmonic structure of the signal, the limit cycle attractor emerging in phase space is not circular but has a more complex structure.
(D) One possible ‘route to chaos’ [30] of a dynamical system is via **subharmonic** oscillation. Subharmonics constitute a special case of frequency modulation (FM) or amplitude modulation (AM), where the relationship between *f_o_* and the modulation frequency (*f*_mod_) is an exact integer (e.g. [37]). The AM example depicted in figure 1D was generated with *f*_mod_ = 50 Hz, *f_o_* = 100 Hz and a modulation extent of 30%, resulting in a time-domain waveform where the amplitude of every odd cycle is slightly lower than that of the even cycles. This constitutes a period-2 subharmonic (or period-doubling) signal (note that more possibilities exist—see [27]). As a result of this signal modulation, additional energy appears between the ‘original’ harmonics (so-called ‘sidebands’)—compare the frequency domain spectrum of figure 1D with that of the periodic case shown in figure 1C. In phase space, the attractor [29]—while technically still a limit cycle—makes two revolutions before repeating. In other words, the cyclical phase space trajectory must complete a motion of 720 degrees (or two cycles).
(E) In contrast to the previous examples, the time-domain waveform in figure 1E appears to be irregular. However, even this **quasi-periodic** case exhibits some inherent form of periodicity, which is revealed by the frequency domain spectrum. The signal contains two superimposed harmonic series with incommensurable fundamental frequencies (labelled *f_o_* and *g_o_*). While in the previous subharmonic example the difference is constituted spectrally by the complex relationship between *f_o_* and *g_o_*, here the frequencies of subharmonic sidebands systematically have simple integer relations to the original signal. In the example shown here, phase space embedding of the quasi-periodic time series reveals a torus attractor [29]. The quasi-periodic signal can emerge in vocal communication in the case of **biphonation**, i.e. in the simultaneous presence of two distinct voice sources, each exhibiting their own harmonic series ([13]; see [38] for review).
(F and G) When considering fully irregular signals, a fundamental distinction needs to be made between **deterministic** (chaotic) and **stochastic** (random) processes, even though voice signals could be a superposition of both. Stochastic processes result in entirely random oscillatory behaviour that is not repeatable and cannot be predicted. In contrast, deterministic processes—arising from simple, low-dimensional systems—are repeatable, provided that the system’s boundary/starting conditions are unaltered [29]. They have an inherent systematic structure that can be described with tools of nonlinear dynamic analysis. While both deterministic and stochastic processes result in irregular (a-periodic) time series, chaos is only constituted by a deterministic process [12]. The example given in figure 1F represents such a deterministic, and thus chaotic, process. It was generated with the logistic map equation *x*[*i* + 1] = *ax*[*i*](1 *x*[*i*]), *a* = 3.6, *x*[0] = 0.5, synthesized with a sampling frequency of 2000 Hz. The first 80 iteration steps are depicted in the left column of figure 1F. The spectrum exhibits a strong sinusoidal component at 1000 Hz, with additional non-harmonic noise components between 0 and 1000 Hz. Phase space embedding results in a simple parabolic attractor (a special form of the *strange attractor* that is typical for chaotic systems [29]), highlighting the non-repeating, deterministic nature of the emerging time series. In contrast, figure 1G shows a stochastic example. The time series was generated by taking the spectral information of the logistic map example above (figure 1F), randomizing all phases and then converting back to the time domain via an inverse Fourier transform. Consequently, the frequency spectrum (middle column), which only depicts the amplitude and not the phase information (a typical practice in voice science), looks identical to that of the logistic map example in figure 1F. The stochastic—and thus random—nature of the example is also not discernible from the time series itself (figure 1G, left panel), but only becomes apparent via phase space embedding. There, no distinct trajectory appears, but the data points assume random values centred at the average amplitude deflections of the time series. This example thus provides an important insight: time series inspection and spectral analysis—as is often done in bioacoustic research—are, strictly speaking, insufficient to distinguish between deterministic (chaotic) and stochastic (random) processes. Rather, analysis methods from nonlinear dynamics are required to reliably achieve this (e.g. [39]).

In nonlinear mathematics, a bifurcation is defined as the abrupt and quantal change of a dynamic system from one oscillatory state to another, brought about by only a small change of boundary conditions or parameter values [40]. Bifurcations can thus be constituted of abrupt changes between any of the seven prototypical oscillatory states described in figure 1 (excluding the ‘sinusoidal’ scenario depicted in figure 1*b*, which represents an artificial scenario), and even changes within one prototypical state (e.g. from a low-frequency to a high-frequency periodic oscillation, as found in yodelling or ‘voice breaks’ of adolescent male humans; [41])—see figure 2. This definition of a bifurcation from nonlinear dynamics is wider than that which is typically considered in texts of animal bioacoustics. For instance, it also includes voice onset or offset. This event is known as a ‘Hopf’ bifurcation [42], i.e. the change from stasis (no phonation) to stable oscillation or vice-versa. In that sense, every vocalization would constitute a bifurcation and thus an NLP, suggesting that perhaps a revision of the terminology used in bioacoustics is needed. Bifurcations can also result in sudden shifts (upwards or downwards) in *f_o_* known as **frequency jumps** [41,43]. It is, however, important to notice that no abrupt change of oscillatory state is automatically a bifurcation in the sense of nonlinear dynamics. In particular, oscillatory state changes brought about by rapid muscular activations, leading to variations of the biomechanical properties of the sound generator may not qualify as bifurcations (see [44]).

Chaotic behaviour and bifurcations typically arise in systems with coupled oscillators, and the voice production apparatus constitutes exactly such a system (but note that deterministic chaos can also occur in simple systems; e.g. figure 1F). The voice source is typically constituted by one or more pairs of oscillating tissues, interacting with the pulmonary air stream. Each of the oscillating components (like the vocal folds, ventricular folds or vocal membranes) has its own set of natural frequencies (termed ‘eigenfrequencies’) that are determined by biomechanical properties and muscular forces. The voice source oscillators are acoustically and aerodynamically coupled to the downstream (in mammals: supraglottal) and the upstream (subglottal) vocal tracts, constituted by pulmonary airways. Vocal tracts have their own geometry (based on anatomical variation) and thus their particular natural frequencies or resonances (often confused with the term ‘formants’ [45]). All these components are in constant negotiation with each other. When entrainment—when two oscillators have (nearly) the same period—of the respective natural frequencies is possible, a stable oscillatory regime may occur (which could be constituted by any of the prototypical states defined in figure 1B–F; see also [20]). However, because a coupled system typically provides more possibilities for entrainment, different oscillatory states are possible at any given time, thus facilitating the emergence of bifurcations and chaotic behaviour. Together, the irregular signals resulting from the oscillatory states described above (biphonation, subharmonics, deterministic chaos and frequency jumps) are referred to as NLP.

### (a) Scoping review

Perceptually harsh and chaotic acoustic features were historically considered as non-adaptive features of vocal communication, or pathological by-products. However, NLP are extremely common in mammalian vocalizations, and increasing evidence suggests that these irregularities may have evolved to fulfil specific socio-ecological functions, such as capturing listeners’ attention or conveying information about emotional status, body size, social dominance or pain [46–49]. Over the last 20 years, advances in our understanding of how NLP might be relevant to non-human animal vocal communication [18], along with increasingly sophisticated analytical tools [27], have led to an increase in interest in this topic, as highlighted by this special issue. Here, we investigate how research has progressed since the earliest papers on NLP in mammalian vocal communication from the early 1960s.

The scoping review aims to

1. Examine research trends in the study of NLP in mammalian vocal communication i.e. the number of papers published over time; the authors and journals publishing NLP research; the taxonomic groups studied; and the methods used to identify or analyse NLP.
2. Contextualize the findings of NLP research within the framework of Tinbergen’s four questions [50]; i.e. what is their adaptive value (function)?; how do they develop during the lifetime of individuals (ontogeny)?; how are NLP produced (mechanism)?; and how do they evolve (phylogeny)?

## 3. Methods

### (a) Literature search

We carried out a comprehensive search of the published NLP literature on 10 May 2024, including any paper that directly recorded data on NLP, tested responses to NLP, investigated the production of NLP or carried out acoustic analysis of NLP. We only considered papers collecting primary data on mammalian species.

We used Google Scholar, Scopus and Web of Science to find relevant publications. This combination of Web of Science and Google Scholar has been shown to provide good coverage of systematic reviews [51], and Scopus has also recently been recommended as an effective search system [52]. We used two sets of keywords: ‘modern’ NLP terms and ‘classic’ NLP terms, as described below. We used the following Boolean phrase to search for the modern NLP terms: mammal* AND nonlinear-phenomena* OR biphon* OR chao* OR subharmonic* OR frequency-jump* AND vocal* OR call*. In order to capture the fullest amount of literature possible on mammalian NLP, we extended the search to terms that were more classically used to describe calls with deterministic chaos using the Boolean phrase: mammal* AND vocal* OR call* AND non-periodic* OR aperiodic* OR nois* OR harsh* OR atonal* OR broadband*. All returned citations were considered and evaluated manually (879 in total, before removing papers not fitting with the requirements above). ‘Grey’ literature was excluded (i.e. non-peer reviewed journal papers).

Finally, to compare the number of results from the NLP literature searches described above with the number of papers investigating vocal communication in mammals more generally, we ran a search using the Boolean phrase: mammal* AND vocal* OR call*. We examined the abstracts of all these papers for relevance and removed inappropriate articles (i.e. those focusing on non-mammalian taxa or human vocalization).

### (b) Data extraction

We compiled a database for all papers returned by our searches, including publication year, journal name and author names. We also noted the order, family, genus and species studied in each paper, using the information given within the paper. We divided the papers by study approach into (i) descriptive studies, in which the presence of NLP was simply described (e.g. presence in a species vocal repertoire); and (ii) empirical studies, in which NLP were analysed statistically (e.g. testing hypotheses relating to NLP). The methods used within each paper were noted, including the use of audio recordings, behavioural observations, playback studies, physiological or anatomical measurements, comparative studies, case studies and methodological studies (i.e. research considering how to study and analyse NLP). These methods were not mutually exclusive, with a study potentially using multiple methods (e.g. an anatomical study may have also used audio recordings). The methods used to identify NLP were recorded, noting whether they used spectrographic analysis (i.e. visual inspection of spectrogram) alone, or in addition to conventional analyses (i.e. harmonic-to-noise ratio (HNR) and Wiener entropy) or nonlinear analyses (i.e. phase space reconstruction). Finally, we considered how each study aligned with Tinbergen’s questions [50], noting how the findings of each study related to the adaptive value, ontogeny, mechanisms or evolution of NLP (studies could be related to more than one question). Only empirical studies were assessed for this analysis, as studies simply describing NLP were often not addressing a specific hypothesis.

## 4. Results

In total, our searches returned 220 papers on NLP in mammals. Of these, 119 papers mentioned the modern NLP terms (biphonation, chaos, subharmonics and frequency jumps), and 101 mentioned the classic terms (non-periodic, aperiodic, noisy, harsh, atonal and broadband). The full dataset can be found in the electronic supplementary material, table S1.

### (a) Research trends in the study of nonlinear phenomena in mammalian vocal communication

**Figure 3.**
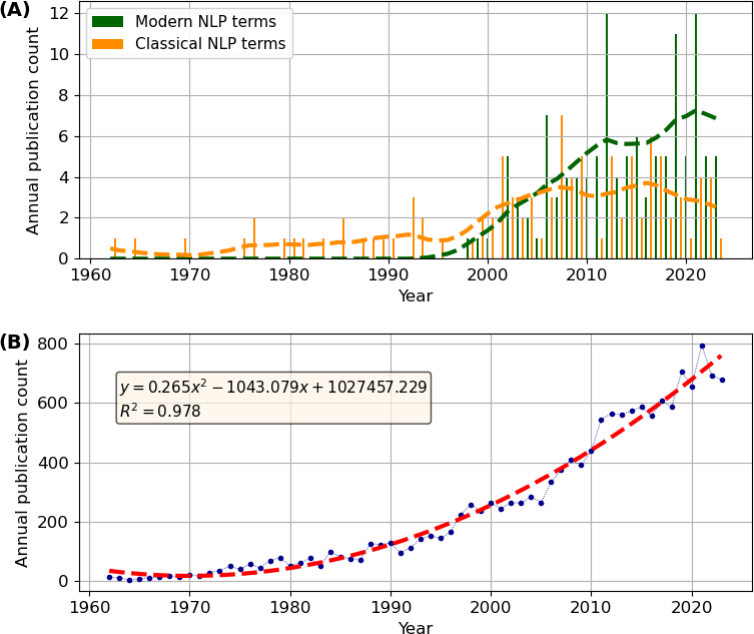
(A) Change in number of mammalian NLP publications with time, from the first publication in 1962 to May 2024. Modern NLP terms (biphonation, chaos, subharmonics and frequency jumps) are plotted in green and classic NLP terms (non-periodic, aperiodic, noisy, harsh, atonal and broadband) are plotted in orange. Vertical lines represent raw frequency data. The trend lines were each computed with 11-point moving average and vertical lines represent raw frequency data. (B) Change in number of all publications on mammalian vocal communication. The trend line was created with a second-order polynomial fit.

The total number of papers published on NLP has increased with time (figure 3A). Papers mentioning classic NLP terms were found as far back as 1962 [53] and increased very slowly, with three papers in the 1960s, four in the 1970s, eight in the 1980s and 10 in the 1990s. This increased to 35 in the 2000s and 32 in the 2010s. Since then, these classic terms appear to be in decline (figure 3A). The number of studies discussing modern NLP terms has increased rapidly since their first use in the mammalian literature in 1998 [14]. There were three papers published in the 1990s, 32 in the 2000s and 60 in the 2010s. The modern terms have thus overtaken the classic terms in their frequency of use and are now used more than twice as often (see figure 3A). This brings the NLP terms used in bioacoustics more in line with the modern terminology used in, e.g. mathematics, physics and engineering, which improves consistency and understanding across fields. However, compared to the more general search for papers on vocal communication in mammals (figure 3B), papers on NLP still represented a very small proportion of the total number of papers overall (mean ± s.d. = 1.62 ± 2.8% of the total number of papers per year; *n* = 220; range = 0–12 papers per year).

The journals that most frequently published papers concerning NLP between 1962 and 2024 were *Bioacoustics*, *Journal of the Acoustical Society of America* and *Animal Behaviour*. The top five journals together covered 43% of all retrieved manuscripts, suggesting that the NLP literature remains concentrated in specialized, field-specific journals, rather than those with broader readerships. A listing of the top 10 journals publishing papers on NLP is presented in electronic supplementary material, table S2. The authors who have, to date, written the most publications on NLP in mammals were IA Volodin and EV Volodina. The top 20 author contributions to the mammalian NLP literature are depicted in electronic supplementary material, figure S1, and the 20 most cited NLP papers on mammals according to Google Scholar are listed in electronic supplementary material, table S3.

In terms of taxonomic coverage, Artiodactyla (*n* = 53), Carnivora (*n* = 52) and Rodentia (*n* = 40) were the orders studied in the greatest number of papers, while Afrosoricida (*n* = 1), Eulipotyphla (*n* = 2) and Dasyuromorphia (*n* = 2) were the least studied (electronic supplementary material, figure S2). Several orders remain completely unstudied and thus did not return any papers in our search. The orders with the widest range of species studied were Rodentia (*n* = 62), Carnivora (*n* = 43) and Primates (*n* = 36), while Sirenia (*n* = 1), Dasyuromorphia (*n* = 1), Afrocsoricida (*n* = 1) and Hyracoidea (*n* = 1) had the fewest species studied (electronic supplementary material, figure S2). A full breakdown of the number of papers by order and family is given in electronic supplementary material, figure S2, and the complete list to level of genus and species are given in electronic supplementary material, table S4.

Of the 220 papers, 59.5% (*n* = 131) were descriptive, simply identifying the use of NLP in the repertoire of a given species, while 40.5% (*n* = 89) took an empirical approach to testing hypotheses in relation to NLP (electronic supplementary material, table S1). Various methods were used across the papers, many of which utilized more than one technique. Studies focusing on audio analysis of NLP were the most common (*n* = 206), and this was also a method used in all other studies. Some studies used behavioural observations to determine contexts for NLP use (*n* = 32). Less common types of study were those using playback experiments (*n* = 23), comparative studies between species or populations (*n* = 18), physiological and anatomical correlates of NLP, such as heart rate or the vocal production mechanisms of NLP (*n* = 17), and ontological studies of NLP call development (*n* = 13). Two studies were methodological, testing new techniques in analysing deterministic chaos and the use of HNR as a means of describing call harshness. One case study was found documenting the vocal changes in a kitten recovering from craniocerebral trauma.

Several analytical methods were used to identify NLP in mammalian vocalizations. Studies investigating deterministic chaos (*n* = 72) largely used spectrographic analysis alone for visual identification (*n* = 51). Other methods included the use of acoustic analyses, such as Wiener entropy and HNR (*n* = 13), and nonlinear methods, including phase space reconstruction, surrogate testing, correlation dimension and Poincare sections (*n* = 8). For studies concerning other NLP, only spectrographic analysis was used in all cases (see electronic supplementary material, table S1).

### (b) Contextualizing the findings of nonlinear phenomena research within the framework of Tinbergen’s four questions

Having provided an overview of the NLP literature on mammals to date, we now briefly summarize the findings by asking how the empirical literature has addressed Tinbergen’s four questions [50] to NLP, i.e. What is their adaptive value (function)? How do they develop during the lifetime of individuals (ontogeny)? How are NLP produced (mechanism)? and How do they evolve (evolution)? From our sample of 89 empirical studies, 62.9% of studies consider adaptive value (*n* = 56), 17.9% ontogeny (*n* = 16), 11.2% evolution (*n* = 10) and 11.2% mechanisms (*n* = 10). A full breakdown of studies and the methods used to investigate Tinbergen’s four questions can be found in the electronic supplementary material, table S1.

#### (i) Function

Most of the empirical studies on NLP in mammals have concerned the adaptive value of NLP in calls, which have been hypothesized to relate to signalling identity, emotional arousal, fitness-related socio-sexual traits and gaining attention (e.g. [14,18,46–49,54,55]), as described in more detail below.

The presence of NLP in calls may encode identity, helping to distinguish individuals, groups or species. For example, dholes, *Cuon alpinu*s, produce a biphonic yap-squeak call that strongly relates to individuality, particularly the high-frequency squeak component [56]. Biphonation was also suggested to be a significant factor for determining individuality in cattle, *Bos taurus* [57]. The biphonic calls of killer whales, *Orcinus orca*, are used more frequently when other pods are present and are thought to act as identifiers of pod membership [58,59]. Further research suggests that it is the high-frequency component of these biphonic calls that encodes pod identity [60]. NLP may also be used to distinguish members of other species, as seen in short-finned pilot whales, *Globicephala macrorhynchus*, and Risso’s dolphins, *Grampus griseus*, which alter their direction of movement in response to calls of their common predator *O. orca* [61].

Emotional arousal is also commonly associated with NLP usage [62–64]. Higher levels of arousal have been linked to increases in NLP, particularly subharmonics, deterministic chaos and frequency jumps. This has been observed in infant and adult African elephants, *Loxodonta africana* (infants: [65]; adults: [66]); vervet monkeys, *Chlorocebus pygerythus* [67]; big brown bats, *Eptesicus fuscus* [68]; and infant giant pandas, *Ailuropoda melanoleuca* [69]. In the biphonic whinnies of horses, *Equus caballus*, the lower fundamental frequency was found to encode arousal, while the upper fundamental frequency was linked to valence [46]. However, there have also been studies in which no clear relationship between arousal and NLP has been found. For example, in domestic kittens, *Felis catus,* there was no significant difference in NLP use between high and low arousal contexts [70]. Similarly, yellow-bellied marmots, *Marmota flaviventer*, with higher levels of glucocorticoid metabolites (a physiological measure of arousal) displayed less deterministic chaos in their alarm calls [71]. However, individuals who were less socially connected within the group, and so were less protected when threatened, displayed more deterministic chaos in their alarm calls, which may reflect higher stress levels as a result of isolation [72].

Calls containing NLP may also impact receivers, serving to gain attention in both alarm and mating contexts. Increased NLP in the alarm calls of meerkats, *Suricata suricatta* [73], and yellow-bellied marmots, *M. flaviventer* [74], led to longer habituation times and increased attention from conspecifics. Similarly, NLP in the mating calls of female red deer, *C. elaphus* [49], and the rejection calls of female koalas, *Phascolarctos cinereus* [75], result in increased looking durations towards the caller. The addition of NLP to calls, therefore, may act to gain attention in alarm or mating contexts. Future directions for research in this area could investigate the emotional contagion potential of NLP in alarm calls, in that NLP may serve to elicit higher arousal states in conspecifics, preparing them to react to a potential threat.

There is evidence that usage of NLP may serve to signal fitness-related socio-sexual traits, such as physical condition, fertility or social rank. For example, NLP are produced in intrasexual agonistic interactions between *C. elaphus* stags in which more subharmonics and deterministic chaos are present in roars made in response to larger opponents [76]. It is proposed that NLP may emphasize the low formant frequencies in roars, which serve to suggest a larger body size [77]. Male rock hyraxes, *Procavia capensis*, display more deterministic chaos in their snort calls with increasing body mass and higher social rank [78]. For those of greater body mass, the ability to produce NLP is thought to function as a handicap signal due to the vocal effort involved, communicating their quality to others, while NLP in higher ranking individual’s calls is thought to imply aggression and so deter conflict with others [78]. A relationship between NLP and fitness is seen in common chimpanzees, *Pan troglodytes*, for which increased NLP in pant-hoot calls may signal good physical condition, as they are energetically costly to produce [55]. NLP may also be used to signal fertility, as the chirp calls of fertile female giant pandas, *A. melanoleuca*, were found to be more chaotic than those of non-fertile individuals, with males approaching those calls preferentially [79].

It is of course possible that NLP are non-adaptive and are instead a by-product of other conditions, such as disease, ill-health or individual variation within the vocal production system. For example, an examination of a kitten with craniocerebral trauma noted biphonation, subharmonics and deterministic chaos in its calls, which reduced and eventually disappeared as the kitten recovered [80]. A greater number of subharmonics, biphonated calls and frequency jumps were also recorded in an infant Japanese macaque, *Macaca fuscata*, suffering from a metabolic disease [54]. When yellow-bellied marmots, *M. flaviventer,* were infected with the parasite *Eimeria*, they produced alarm calls containing higher portions of deterministic chaos than healthy individuals [81]. Additionally, NLP have been used to distinguish several voice pathologies in humans [82].

Most of the empirical studies into the adaptive function of NLP used audio analysis to identify or quantify NLP, whereas only a smaller number used an experimental approach. Playback studies provide a useful framework for more directly testing the adaptive value of behaviour and/or the cognitive mechanisms underpinning receiver responses. For example, there are multiple experimental approaches that could be used to test whether NLP encodes individual identity, including natural playbacks, go/no-go experiments, discrimination tasks and habituation/dishabituation [83]. Similarly, playback experiments would provide insight into how emotion and/or fitness-related socio-sexual traits might be encoded in NLP. However, to date the number of such studies has been very limited. They have also been restricted to the use of natural calls to measure animals’ responses and have thus been unable to disentangle the effects of NLP from those caused by other covarying acoustic features, such as call frequency. Excitingly, ground-breaking tools, including *Soundgen* ([84]; see also [27]), now allow for much more robust investigations into the function of NLP through the creation of synthetic stimuli [85], which we expect will push forward a new understanding of the adaptive function of NLP in animal communication. Linking such experiments with detailed investigations into genetics, physiology, cognition and/or behaviour will allow for even further insights.

#### (ii) Ontogeny

The second largest proportion of studies (17.9%) investigated the development of vocalizations across the lifetime or developmental stages of a species, noting how NLP appear, alter or disappear with age [86]. For example, in North Atlantic right whales, *Eubalaena glacialis*, the type and amount of NLP expressed varies throughout their lives [87]. Calves show the highest levels of deterministic chaos in their calls, which decrease sharply as they become juveniles and disappear entirely when whales are over 25 years old. Subharmonics and biphonation first appear in juveniles and then increase in usage over time. The authors suggest that these changes are likely due to increased vocal control with age. A study examining the development of common marmosets, *Callithrix jacchus*, found that within the first two months of life, infants progress from producing cries, to ‘phee’ calls with subharmonics, and finally into adult phees displaying no subharmonics [88]. Respiratory monitoring and biomechanical models suggested that air pressure and muscle tension increased as calls shifted from infant cries to adult phees. Alongside increased respiratory control, parental feedback was necessary for the calls to properly develop. Similarly, the ultrasonic vocalizations of yellow steppe lemmings, *Eolagurus luteus*, change in their expression of NLP during the first 90 days of life [89]. Biphonation is common at 1−8 days and then disappears from calls over time, while subharmonics and frequency jumps are reduced but remain in adulthood. Grimsley *et al*. [90] noted that mice pups have increasing levels of subharmonics and deterministic chaos in their isolation calls up to adulthood, at which point they quickly disappear. Another study compared the isolation calls of deaf and hearing transgenic mice, finding that calls did not change and still included frequency jumps with age, ruling out auditory experience as a necessary part of vocal development in this species [91]. These findings reflect those of human speech studies, in which infant cries contain deterministic chaos while NLP is extremely rare in adult speech. However, NLP are common in some adult non-verbal vocalizations [13,92–94]. Suggested reasons for the presentation of NLP in early life include the identification of infants or juveniles, maturation of the vocal tract, lack of vocal control or experience or to convey emotional arousal to parents. However, the majority of studies did not test for these functions or the mechanisms by which they change. It also remains unclear why some NLP disappear in adulthood while others remain. Further research is needed to understand how ontogenetic changes in anatomy and neurobiology might link to the production of NLP and the degree to which these differences in NLP production across the lifespan might be adaptive. Comparative analyses might help us understand the evolution of NLP across the lifespan. However, data are very limited for most clades and strongly biased to a small number of mammalian orders, as shown above.

#### (iii) Mechanism

The papers retrieved from our search (*n* = 10) discussed several anatomical and physiological mechanisms underlying the production of NLP, documented in several different species. Some studies were carried out *ex vivo*, using excised larynx experiments to document vocal fold vibration. For example, experiments on Bolivian squirrel monkey*, Saimiri boliviensis boliviensis*, and Peruvian squirrel monkey, *Saimiri boliviensis peruviensis*, larynges identified two nonlinear regimes (biphonation and deterministic chaos) out of four regimes of vocal fold activation [95]. These regimes only occurred at moderate to high subglottal pressures, relating to known calls in their repertoire. In another experiment, the occurrence of periodic, subharmonic and chaotic vibratory regimes were shown for the excised larynx of an African elephant [34]. Here, the authors suggest that these NLP are due to vocal fold dynamics rather than interactions between the vocal folds and the vocal tract [34]. Conversely, frequency jumps in acoustic data from living humpback whales, *Megaptera novaeangliae*, are proposed to be due to coupling between the laryngeal source and resonant cavities within the vocal tract [96]. An examination of the larynx of a dog–wolf hybrid showed higher levels of subharmonics, deterministic chaos and biphonation, which were not present in the dog and wolf individuals [97]. These differences may have been due to a small asymmetry in part of the arytenoid cartilage and the presence of a lip on the vocal folds that was not present in the other individuals. Another anatomical investigation into the larynx and upper tract of male North American wapiti, *Cervus canadensis*, suggested that the lower frequency portion of biphonic calls is produced by the larynx, while the upper frequency portion was suggested to be made by an aerodynamic whistle [98]. By using a biomechanical model of the larynx, another study on *M. novaeangliae* found that frequency jumps and deterministic chaos became stronger with increasing subglottal pressure [99]. Frequency jumps also got larger when the U-fold (homologous to the vocal folds in baleen whales) was thicker and longer, when the volume of the laryngeal sac varied sharply and when the stiffness of the U-fold was asymmetrical. Finally, an *in vivo* study of elicited phonation from stimulation of the cricothyroid muscle in New Zealand white breeder rabbits, *Oryctolagus cuniculus*, noted that increased production of all types of NLP was related to airflow rate [100].

While the vocal anatomy for mammalians is generally similar across species, the mechanisms behind the production of NLP vary across species and can include varying subglottal pressure, airflow rate, vocal fold dynamics and the size and asymmetry of a range of anatomical features [18,19,101–103]. A further understanding of these mechanisms and how and why they differ across species would help advance our understanding of the evolution and ecology of NLP production and its relevance for vocal communication systems.

Whether NLP are typically produced by the voice source alone or via interactions between the source and filter (vocal tract) remains an important open question. Both options are possible, and this has been reasonably well studied in humans, leading to the nonlinear extension of the classic SFT [20,44]. However, source–filter interactions are extremely complicated to study in non-human animals. Excised larynx experiments are typically carried out on the source, detached from the vocal tract, and are thus not suitable for answering such questions. An alternative approach would be to perform whole-head experiments, but to the best of our knowledge, this has only previously been carried out by Ferrein in the eighteenth century [104]. *In vivo* approaches, such as laryngoscopy, are possible in humans but generally only possible with non-human animals under anaesthesia [103]. However, non-invasive approaches, including electroglottography, might provide an exciting avenue for future *in vivo* research into the mechanisms of vocal production in a comparative framework ([25,105]; also see [41]).

#### (iv) Evolution

To date, there have been no macro-evolutionary studies using phylogenetic comparative methods to investigate NLP. However, three studies have compared species and populations and discussed potential drivers of NLP evolution. Bergman et al. [106] investigated the loud calls of Guatemalan black howler monkeys, *Alouatta pigra*, and mantled howler monkeys, *A. palliata*, finding that *A. pigra* display more deterministic chaos in their calls (measured as HNR). They suggest that this difference evolved in relation to differences in their social structure and thus different uses of their loud calls. *A. palliata* live in multimale groups and may call to attract potential mates, while *A. pigra* live in smaller groups, with fewer males, and call more in intrasexual confrontation with others [6,106]. Differences between the loud calls of harvest mice may be driven by habitat differences, as the fully arboreal Mexican harvest mouse, *Reithrodontomys mexicanus*, shows subharmonics in their calls, while terrestrial species did not [107]. A potential reason for these differences may be that subharmonics help to propagate calls in a complex auditory environment, such as the dense forest canopy where conspecifics cannot be easily seen. In these cases, subharmonics could be acting to lower the fundamental frequency of calls. In North Pacific killer whales, *O. orca*, monophonic calls are found to be more diverse between populations than biphonic calls [108]. The authors suggest that as their study population grew there was increased pressure for biphonic calls to remain static as they likely function as group identifiers. Overall, while there have been no phylogenetic studies involving NLP, comparative studies have noted possible sources of evolutionary pressure on various species, such as habitat, social structure and the need to identify individuals, opening potential avenues for larger scale species comparisons in the future. A challenge to such work is the notable bias in the taxonomic coverage of NLP studies in mammals.

## 5. Conclusions and outlook

NLP is a complex yet ubiquitous aspect of animal vocalization that has been of increasing interest to bioacousticians in recent decades. Research into NLP in mammals has progressed significantly in the last 20 years, providing valuable insights into the adaptive function, ontogeny, production mechanisms and evolution of NLP across a wide variety of species. Tinbergen’s questions offer us a framework for future investigations into NLP, highlighting that while much research has been conducted on adaptive function, our understanding is still lacking in terms of ontogeny, mechanisms and evolution. With modern terminology and the use of novel tools for analysis and playback studies, NLP research can become more cohesive and impactful, fostering better communication between bioacousticians on the topic. The study of NLP is an ongoing development, and the already existing body of literature is a testimony to the importance of NLP in animal vocalizations. We look forward to a new age of NLP research, which we anticipate will lead to a paradigm shift in our understanding of vocal communication in mammals.

## Data Availability

All raw data are provided in the supplementary materials [109].
